# Analysis of Intrinsic Breast Cancer Subtypes: The Clinical Utility of Epigenetic Biomarkers and *TP53* Mutation Status in Triple-Negative Cases

**DOI:** 10.3390/ijms232315429

**Published:** 2022-12-06

**Authors:** Ieva Sadzeviciene, Kristina Snipaitiene, Asta Scesnaite-Jerdiakova, Kristina Daniunaite, Rasa Sabaliauskaite, Aida Laurinaviciene, Monika Drobniene, Valerijus Ostapenko, Sonata Jarmalaite

**Affiliations:** 1 Institute of Biosciences, Life Sciences Center, Vilnius University, Sauletekio Ave. 7, LT-10257 Vilnius, Lithuania; 2 National Cancer Institute, Santariskiu St. 1, LT-08406 Vilnius, Lithuania; 3 National Center of Pathology, Affiliate of Vilnius University Hospital Santaros Clinics, P. Baublio St. 5, LT-08406 Vilnius, Lithuania

**Keywords:** breast cancer, triple-negative breast cancer, DNA hypermethylation, *TP53*, *FILIP1L*, *MT1E*

## Abstract

This study aimed at analyzing the DNA methylation pattern and *TP53* mutation status of intrinsic breast cancer (BC) subtypes for improved characterization and survival prediction. DNA methylation of 17 genes was tested by methylation-specific PCR in 116 non-familial *BRCA* mutation-negative BC and 29 control noncancerous cases. At least one gene methylation was detected in all BC specimens and a 10-gene panel statistically significantly separated tumors from noncancerous breast tissues. Methylation of *FILIP1L* and *MT1E* was predominant in triple-negative (TN) BC, while other BC subtypes were characterized by *RASSF1, PRKCB, MT1G, APC*, and *RUNX3* hypermethylation. *TP53* mutation (*TP53-mut*) was found in 38% of sequenced samples and mainly affected TN BC cases (87%). Cox analysis revealed that TN status, age at diagnosis, and *RUNX3* methylation are independent prognostic factors for overall survival (OS) in BC. The combinations of methylated biomarkers, *RUNX3* with *MT1E* or *FILIP1L*, were also predictive for shorter OS, whereas methylated *FILIP1L* was predictive of a poor outcome in the *TP53-mut* subgroup. Therefore, DNA methylation patterns of specific genes significantly separate BC from noncancerous breast tissues and distinguishes TN cases from non-TN BC, whereas the combination of two-to-three epigenetic biomarkers can be an informative tool for BC outcome predictions.

## 1. Introduction

In 2020, breast cancer (BC) was the leading cause of women’s cancer worldwide, while the mortality from BC was in fifth place [1]. Triple-negative (TN) BC accounts for approximately 10–15% of all diagnosed BC and, in comparison to estrogen- and progesterone receptor-positive (i.e., hormone receptor-positive, ER+ and PR+, respectively) BC cases have the most aggressive course of the disease and the worst prognosis [2]. In contrast to the hormonal or HER2 (human epidermal growth factor receptor) positive BC, the specific molecular pathophysiology of TN BC remains poorly understood, resulting in a lack of efficient target therapies [3].

Nowadays, immunohistochemistry is mainly used for BC subtype classification but the addition of genetic and epigenetic biomarkers could increase the sensitivity and specificity of disease diagnosis, prognosis, and prediction of treatment outcome. Because BC is a multiform disease and there is no particular cause or gene mutation to lead the breast cell to cancer, it is important to find an informative biomarker system to identify and predict the disease development and response to treatment.

*TP53* mutation is the most frequent mutation in invasive BC, occurring in about 30–35% of all cases, and approximately 80% of them are found in TN tumors [4]. *TP53* is activated in response to DNA damage, oncogene activation, hypoxia, or cellular stress and is involved in cell-cycle arrest, apoptosis, senescence, or autophagy modulation processes [5]. Therefore, the *TP53* mutation is a marker for resistance to chemotherapy and radiotherapy and, consequently, a poor prognosis of the disease [6].

DNA methylation is a stable and reversible molecular alteration of DNA that changes the gene expression patterns but does not affect DNA sequences. In humans, methylation of cytosine occurs mainly in CpG dinucleotides (75%) [7]. Normally, unmethylated CpG islands in cancer cells may become methylated, resulting in the silencing of important genes involved in various cellular processes, including cell-cycle regulation, DNA repair, cellular homeostasis, apoptosis, cell adhesion, and invasion. Epigenetic change is an early event in tumor formation and also can be the second “Knudson’s hit” in cell tumorigenesis [8]. DNA methylation biomarkers can increase the accuracy of the disease diagnosis, prognosis, and prediction of treatment outcome; therefore, for our study biomarkers, which are involved in cell cycle regulation (*P14^ARF^,* later *P14* and *P16^INK4A^,* and later *P16*), cellular signaling pathways (*ESR1* (two different regions)*, RARB, RASSF1,* and *PRKCB*), programmed cell death (*DAPK1*), cell adhesion (*APC*), angiogenesis process (*ADAMTS12* and *FILIP1L*), maintenance of genome stability (*MGMT*), xenobiotic metabolism (*GSTP1*), heavy metal binding (*MT1E, MT1F,* and *MT1G*) and gene expression (*RUNX3*), were selected.

The aim of the present study was to investigate the DNA methylation pattern of intrinsic BC subtypes, especially of the TN BC, in association with *TP53* mutation status, and to identify biomarkers for the disease outcome predictions.

## 2. Results

### 2.1. DNA Methylation Spectrum

**Figure 1:**
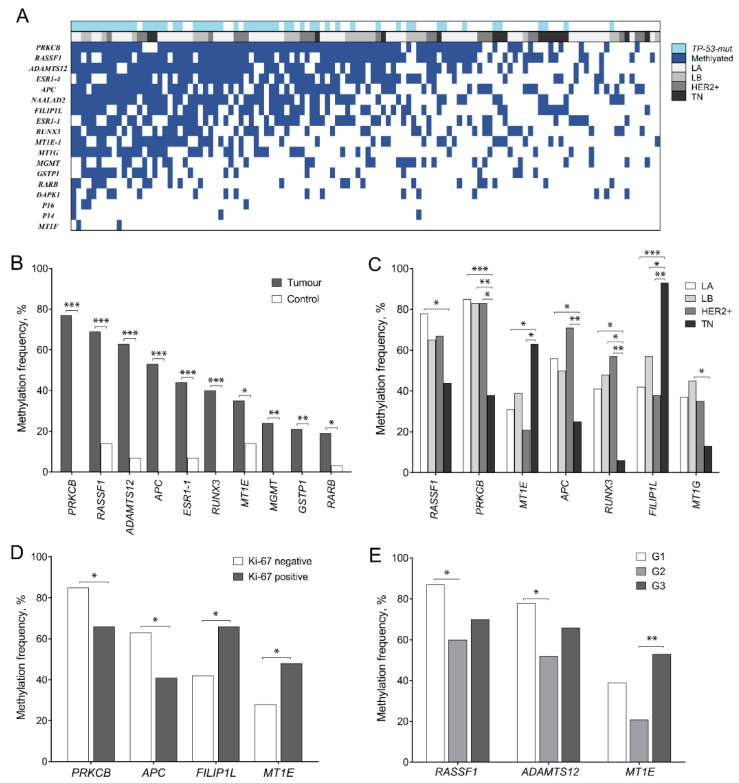
Promoter methylation frequencies of the analyzed genes in breast cancer (BC): heatmap of entire BC cohort (**A**), tumors vs. controls (**B**), BC cases according to the intrinsic subtypes (**C**), tumor Ki-67 status (**D**), and differentiation grade (**E**). G1—good, G2—moderate, G3—poor differentiation grade; HER2+−HER2 positive and Luminal B HER2 positive; LA—Luminal A; LB—Luminal B; TN—triple-negative BC; *TP53-mut*—*TP53* mutated; * *p* < 0.05; ** *p* < 0.01; *** *p* < 0.001.

DNA methylation status of selected tumor suppressor genes (*P14, P16, MGMT, RARB, RASSF1, DAPK1, GSTP1, ESR1-1, ESR1-4, PRKCB, MT1E, MT1F, MT1G, APC, ADAMTS12, RUNX3, NAALAD2,* and *FILIP1L*) was analyzed in 116 BC and 29 control samples. At least one methylated gene was detected in every sample (Figure 1A).

In BC samples, promoter hypermethylation was the most frequently detected in *PRKCB* (86/111, 77%), *RASSF1* (79/115, 69%), *ADAMTS12* (67/107, 63%), *APC* (61/115, 53%), and *RUNX3* (46/114, 40%) genes and significantly differentiated BC from the control group (0%, 14%, 7%, 0%, and 0%, respectively; all *p* < 0.0001). Hypermethylation of *ESR1-1, RARB, GSTP1, MGMT*, and *MT1E* genes was detected in 19–44% of BC samples and the changes were significantly different as compared to the controls (0–14%, all *p* < 0.05; Figure 1B). Hypermethylation frequency of *MT1F, P14, P16, DAPK1, MT1G, FILIP1L, NAALAD2*, and *ESR1-4* was quite variable (2–57%) and the differences from controls (methylated 0–64%) were not statistically significant (*p* > 0.05) (The detailed information on biomarker sensitivity and specificity is given in Supplementary Table S1).

Methylation of *FILIP1L* and *MT1E* was predominant in TN BC, while other subtypes were characterized by frequent methylation of *RASSF1, PRKCB, MT1G, APC*, and *RUNX3* (*p* < 0.05; Figure 1C). Analysis of separate molecular biomarkers confirmed the same changes as in subtype analysis according to hormone receptor status: ER+ cases more frequently had methylated *PRKCB* (86% vs. 40%) and *RUNX3* (46% vs. 18%) than ER negative, while *FILIP1L* promoter hypermethylation was dominating in ER and PR negative BC (84% vs. 45% and 76% vs. 47%, respectively; in all cases *p* < 0.05). In addition, *MT1E* and *FILIP1L* were more frequently hypermethylated in Ki-67+ cases (48% vs. 28% and 66% vs. 42%; *p* < 0.05); however, no statistically significant association with the HER2 receptor was detected (Figure 1D).

The comparison of promoter hypermethylation between ductal and lobular BC revealed more frequent *MT1E* methylation in ductal BC (40% vs. 7%; *p* = 0.018). Tumor stage analysis showed that the *P16* promoter was methylated only in stage T2 tumors (8% vs. 0%; *p* = 0.041), while promoter hypermethylation of *RUNX3* was significantly more common in cases with affected nodes (52% vs. 31%; *p* = 0.032). Analysis of associations between biomarkers and tumor grading revealed statistically significant differences in promoter hypermethylation of *RASSF1, ADAMTS12* (G1 vs. G2), and *MT1E* (G2 vs. G3) (*p* < 0.05; Figure 1E). Promoter methylation frequencies of *ADAMTS12* (64%), *ESR1-4* (53%), *MT1E* (40%), and *DAPK1* (15%) were higher in the younger patients’ group (*p* < 0.05). Detailed methylation distribution between subtypes and other clinical–pathological variables is provided in Supplementary Table S1.

### 2.2. TP53 Mutation Spectrum

**Figure 2:**
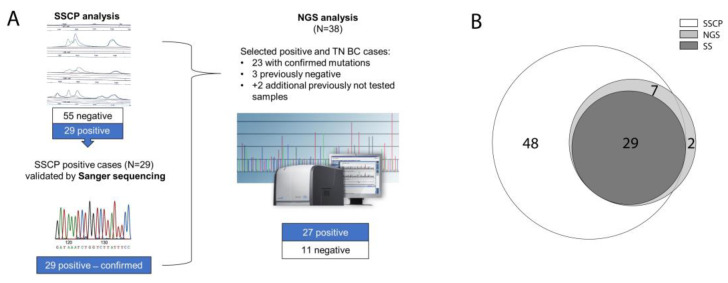
The schematic view of *TP53* sequencing analysis: the workflow (**A**), and Venn diagram depicting samples overlapping between different methodologies for mutation detection (**B**); NGS—next-generation sequencing; SS—Sanger sequencing; SSCP—single-strand conformation polymorphism.

In total, 86 tumors were analyzed for *TP53* mutations (*TP53-mut*), out of which sequence alterations were identified in 33 cases (38%,), while 53 BC had *wild-type TP53* (62%, *TP53-wt*). After 84 samples were analyzed by using the single-strand conformation polymorphism (SSCP) method, 29 *TP53-mut* cases were detected and further validated by Sanger sequencing (SS). Thirty-eight samples were selected for more detailed analysis using next-generation sequencing (NGS), out of which two samples were not previously analyzed either by SSCP or by SS (Figure 2A,B). Three samples, previously determined as negative by SSCP and SS methods were identified as *TP53-mut* positive by NGS analysis.

Out of 33 *TP53-mut* BC cases, pathogenic *TP53* sequence alterations were detected in 27 BC, 78% (21/27) of which occurred in the DNA binding domain, and 22% (6/27) in introns. According to the mutation type, about a half (52%, 14/27) of pathogenic mutations were missense, 19% (5/27) splice, 11% (3/27) frameshift, 11% (3/27) nonsense, and 4% (1/27) intronic. Out of pathogenic mutations: 26% were AT:GC; 19% were GC:AT at CpG sites, and 15% were not at CpG sites; 11% were GC:TA. Deletions in the studied *TP53* gene region were quite common and accounted for 15% of all alterations (range 1–23 nt). The largest, 23-nucleotide (nt) deletion g.7578546_7578568del (23 nt deleted) was found in the fourth intron by the NGS method and affected the splicing site. The detailed *TP53* mutation data are provided in Supplementary Table S2.

### 2.3. TP53 Mutations Predominate in TN BC Subtype

**Figure 3:**
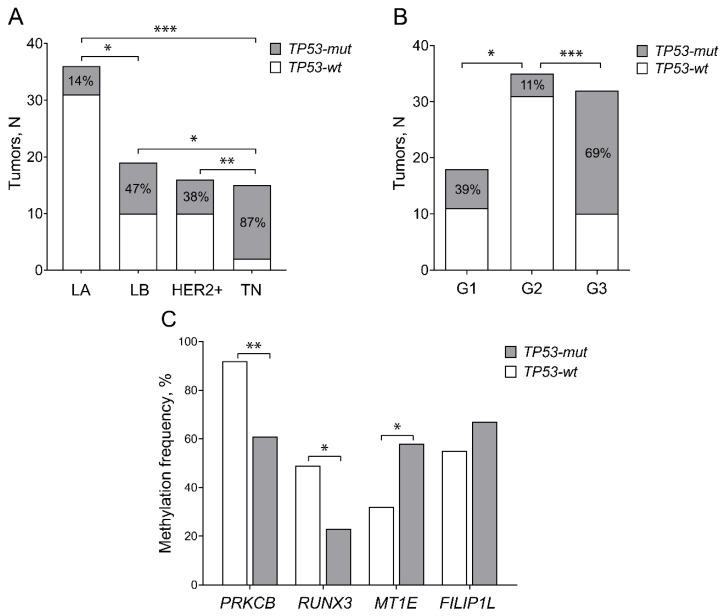
*TP53* mutation distribution among breast cancer (BC) patients grouped according to intrinsic subtypes (**A**), tumor differentiation grades G (**B**), and methylation status of analyzed genes (**C**). G1—good, G2—moderate, G3—poor differentiation; HER2+—HER2 positive and LB HER2 positive; LA—Luminal A; LB—Luminal B; TN—triple-negative BC; *TP53-mut*—*TP53* mutated and *TP53-wt*—*wild type TP53* gene status; * *p* < 0.05; ** *p* < 0.01; *** *p* < 0.001.

*TP53-mut* cases were frequently negative for ER and/or PR (79%, *p* < 0.0001 and 57%, *p* = 0.006, respectively); consequently, the *TP53* gene alterations were predominant in the TN BC subtype (81%) and were relatively rare in HER2+ (26%), LB (39%), and LA (9%) BC subtypes (all *p* < 0.05; Figure 3A). Additionally, *TP53-mut* tumors had a significantly higher expression of Ki-67 (*p* < 0.0001); however, no associations were found with HER2 (*p* > 0.05).

Analysis of other clinical–pathological variables revealed that poorly differentiated BC tumors (G3; 67%) were more frequently mutated than moderately differentiated tumors (G2; 11%; *p* < 0.0001; Figure 3B). The detailed comparison of clinical–pathological characteristics of patients according to *TP53* mutation status is presented in Table 1. According to *TP53* mutation status, almost two times more frequent methylation of *MT1E* was identified in *TP53-mut* than in *TP53-wt* BC cases (58% vs. 32%; *p* = 0.024), whereas methylation frequencies of *PRKCB* and *RUNX3* were significantly higher in the *TP53-wt* subgroup (92% vs. 61%, *p* < 0.001, and 49% vs. 23%, *p* = 0.021, respectively; Figure 3C).

### 2.4. Prediction of Overall Survival

Univariate and multivariate Cox proportional hazards regression analyses were performed to analyze the associations between the biomarkers and the overall survival (OS) of BC cases. In univariate analysis, older age and TN subtype were significantly associated with shorter OS (*p* < 0.05). Out of the analyzed genetic biomarkers, only the hypermethylation of *FILIPL1* tended to be associated with OS (HR = 3.3, 95% CI 0.9–12.0, *p* = 0.067; Table 2; presented are only the genes demonstrating HR > 1.0).

In multivariate analyses, by applying the inclusion criteria of HR>1.0 and p<0.2, the joint analysis consisting of *FILIP1L, P16, RUNX3*, age, G, N status, and TN subtype (underlined in Table 2) revealed that age (HR = 1.07, 95% CI (1.02–1.12); *p* = 0.010), TN status (HR = 13.92, 95% CI (2.97–65.20); *p* = 0.010), and *RUNX3* methylation (HR = 4.64, 95% CI (1.15–18.75); *p* = 0.032; Table 2) are independent prognostic factors for OS.

**Figure 4:**
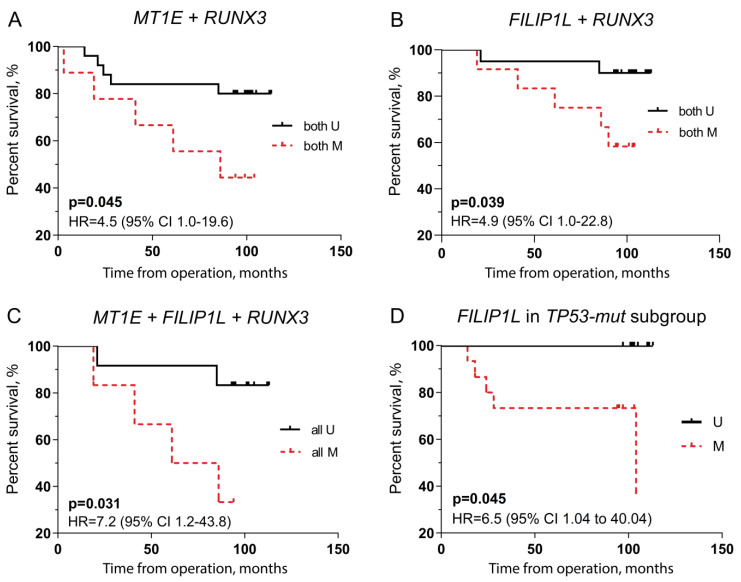
Overall survival prediction using *RUNX3* in combination with *MT1E* (N = 9 vs. 27; corrected *p* = 0.180) (**A**) and *FILIP1L* (N = 14 vs. 22; corrected *p* = 0.156) (**B**), three biomarkers combination (N = 6 vs. 13; corrected *p* = 0.124) (**C**), and *FILIP1L* in the *TP53-mut* BC subgroup (N = 15 vs. 8; corrected *p* = 0.180) (**D**). CI: confidence interval; HR: hazard ratio; M—methylated and U—unmethylated status of biomarker; *TP53-mut*—*TP53* mutated.

In the Kaplan–Meier survival analysis, various combinations of biomarkers were predictive for the outcome: *RUNX3* combinations with *MT1E* or *FILIP1L* (*p* = 0.045 and *p* = 0.039, respectively; Figure 4A,B) or all three biomarkers also significantly predicted the poor outcome (*p* = 0.031; Figure 4C). In addition, *FILIP1L* methylation was predictive of poor outcomes in the *TP53-mut* subgroup (*p* = 0.045; Figure 4D).

## 3. Discussion

The heterogeneity of BC is reflected by gene expression patterns known as intrinsic BC subtypes, which nowadays are classified according to IHC biomarkers; however, these subtypes further vary in the abundance of genetic mutations and epigenetic alterations. The luminal and HER2 receptor-expressing BC can be treated using modern targeted therapy, unlike the TN subtype, which is the most heterogeneous group of BC lacking efficient diagnostic and treatment modalities [9]. Early BC diagnostics, especially the TN subtype, could improve BC survival rates; therefore, the traditional IHC-based diagnostic methods may benefit from supplementing by genetic and epigenetic biomarkers.

DNA methylation changes of selected genes were identified in all BC, and the methylation pattern of 10 out of 17 tested genes statistically significantly separated tumors from noncancerous breast tissues. DNA methylation in some of the genes was predominant in less aggressive G1 (*RASSF1* and *ADAMTS12*) or Ki-67 negative (*PRKCB* and *APC*) tumors, indicating an early occurrence of epigenetic events. Moreover, specific DNA methylation patterns were characteristic of intrinsic BC subtypes in our and other studies [10,11]. For instance, luminal subtypes harbor subtype-specific methylation biomarkers like *RASSF1*, *GSTP1*, *APC*, *ADAMTS12*, and *PRKCB* [10,12]. In our study, the biomarker set of *PRKCB, RASSF1,* and *APC* was found to be hypermethylated in a majority of BC samples with the highest specificity to BC and was specific to hormonal and HER2+ BC subtypes as well, significantly distinguishing from the TN BC subtype. On the contrary, studies show that TN tumors have fewer DNA methylation changes than non-TN [13] but TN BC is the most heterogenous BC intrinsic subtype, molecularly subcategorized into smaller subgroups [14]. TN BC is diverse and difficult to study and therefore there is only a handful of studies assigning specific biomarkers to TN BC [15,16]. In our study, the increased DNA methylation rate of *MT1E* and *FILIP1L* significantly distinguished the TN BC subtype from luminal and HER2+ BC subtypes with twice higher hypermethylation frequency, and both showed an association with Ki-67 expression. Differences in DNA methylation patterns between and even within BC intrinsic subtypes demonstrate high biological variability of these tumors and show the need for further subclassification of BC for cost-effective treatment personalization.

Because of the high heterogeneity and limited treatment options, the survival of TN BC is well known to be the lowest; however, the complete picture of molecular pathways affected in TN BC remains unclear and targets for efficient treatment are yet to be found. Some of the studies reveal the significance of epigenetic factors in TN BC pathogenesis [17]. It has been demonstrated [18] that the hypomethylated profile TN has a better survival than hypermethylated, however, survival of TN BC cases of the medium methylated cluster was shown to be the worst. Genes hypermethylated in TN BC are involved in various cellular pathways and could be used to predict survival outcomes and response to treatment [15,16]. In our study, despite relatively low DNA methylation frequencies detected in TN BC, the hypermethylation of *RUNX3*, *MT1E*, and *FILIP1L* was highly specific to this subtype and associated with a shorter OS when analyzed alone (*RUNX3*) or in combinations (*RUNX3*, *FILIP1L*, and *MT1E*). *RUNX3* encodes a tumor suppressor which regulates cell growth, survival, differentiation, angiogenesis, and invasion [19]; *FILIP1L* is a protein that inhibits metastases and chemoresistance [20]; *MT1E* is a cytoskeleton-modifying protein, involved in cell migration and invasion [21]. All these newly identified biomarkers of TN BC demonstrate the potential to accompany classic diagnostic methods and become a part of companion diagnostics for novel therapies, including combined treatment schemes that involve epigenetic drugs.

The TN BC subtype differs from luminal and HER2+ subtypes in a genetic and epigenetic manner. *TP53* mutation is found in approximately 80% of TN BC cases [4,22] and is associated with poor prognosis [6]. Similarly, in our study, *TP53* mutation predominantly occurred in TN BC (87%) but was rarely observed in other BC subtypes. In addition, our research showed that in TN BC, more than two-thirds of *TP53* mutations occurred among poorly differentiated tumors and were associated with higher Ki-67 expression. In our study, more than half of *TP53* alterations were missense mutations, which, according to Sousse and colleagues [23], result in a stable p53 protein that lacks its specific DNA-binding activity, accumulates in the cellular nucleus where, by interacting with oncogenes, causes cell transformations [24]. Although *TP53* mutations are predominant in the TN subtype, they can also be associated with ER+ patients’ survival, affecting their response to endocrine therapy [25]. Taken together, *TP53* is an important player in breast carcinogenesis and a significant target for specific treatment development.

Despite this study being performed by investigating both genetic and epigenetic alterations of BC, several shortcomings can be discussed. The study cohort included all BC subtypes and the TN BC part comprised only 14% of all cases; therefore, further analysis of TN BC-specific biomarkers should be extended to a larger independent TN BC cohort. As this study was started some years ago, more extensive use of the NGS method now is possible and looks more informative for *TP53* mutations analysis. While different studies show that *TP53* mutations could be associated with a poor, good, or neutral outcome, mainly, *TP53-mut* tumors are associated with worse OS [26]; however, in the current study, a *TP53-mut* association with worse OS was not demonstrated. Additionally, follow-up data were missing for some patients, which could have affected the OS statistics.

## 4. Materials and Methods

### 4.1. Patients and Samples

In total, 116 BC patients and 29 control cases with fibroadenoma (all white Caucasian race females) treated at the National Cancer Institute of Lithuania enrolled in the study in 2007–2009. The Bioethics Committee approved the study (2007-08-03 No. 33) and informed consent was obtained from every case before entering the study. All investigated BC cases were *BRCA*-negative non-familial cases. The mean age of BC patients was 57 years (range 27–84 yrs.), and 42 years for controls (range 20–62 yrs.); *p* < 0.05. All patients were diagnosed with invasive BC of early stages T1 (n = 63) and T2 (n = 53). The analyzed BC types were ductal (n = 101), lobular (n = 13) and apocrine (n = 2) breast carcinomas. The intrinsic subtypes of BC were identified based on the IHC status of pathology biomarkers: estrogen (ER) and progesterone (PR) receptors, human epidermal growth factor receptor-2 (HER2), and marker of tumor proliferation (Ki-67). Ki-67 cut-off in our study was 15%; therefore, >15% was considered as Ki-67 positive and, on the contrary, <15% of Ki-67 was considered as Ki-67 negative. In addition, 47% were of luminal A (LA, n = 55), 21% of luminal B (LB, n = 24), 18% were HER2+ (n = 21, out of which 16 and 5 cases were LBHER2 and HER2, respectively), and 14% were triple-negative (TN, n = 16) BC cases. Follow-up data were available for 78 of 116 (67%) BC cases and the average follow-up time was 91 (range 3–113) months. Out of 78 patients whose outcomes were known, 21 cases were deceased, 1 relapsed, and 56 were in remission. Detailed information on demographic and clinical–pathological variables according to intrinsic BC subtypes is provided in Table 3.

### 4.2. DNA Extraction

DNA was extracted by the standard phenol-chloroform purification and ethanol precipitation method and using ZR Viral DNA/RNA Kit™ (Zymo Research, Irvine, CA, USA) from fresh-frozen and ground tumor tissue specimens (n = 79) after digestion with proteinase K and from formalin-fixed paraffin-embedded (FFPE) tissues (n = 45) after the deparaffinization. DNA concentration and quality parameters were evaluated spectrophotometrically by using NanoDrop^TM^ 2000 (Thermo Scientific, Thermo Fisher Scientific (TFS), Waltham, MA, USA).

### 4.3. DNA Methylation Assay

Isolated DNA (400 ng) was first modified with sodium bisulfite using EZ DNA Methylation™ Kit (Zymo Research, Irvine, CA, USA) according to the manufacturer’s recommendations. For DNA methylation assessment, the pairs of primers specific to methylated (M) and unmethylated (U) sequences within the 5‘region of *P14*, *P16*, *MGMT, RARB, RASSF1, DAPK1, GSTP1, ESR1* (two 5′ regions of the *ESR1* gene, one in promoter region and one intragenic sequence, were included into this study and marked as *ESR1-1* and *ESR1-4*, respectively), *PRKCB, MT1E, MT1F, MT1G, APC, ADAMTS12*, and *RUNX3.* Genes were designed using Methyl Primer Express v1.0 software (Applied Biosystems (ABI), TFS) or selected based on BC specificity and diagnostic and/or outcome prediction capabilities from our previous studies [27,28,29] (see Supplementary Table S3). Methylation-specific PCR (MSP) mix of the final volume of 25 µL contained 10 ng of bisulfite-modified DNA template, PCR buffer, 1.6 mM of each dNTP, 2.5 mM of MgCl2, 1 µM of each primer, enhancer, and 0.5 U of Gold polymerase (ABI, TFS). PCR was performed in a thermocycler at the conditions provided in Supplementary Table S4. Each PCR run was performed by using two kinds of DNA methylation controls, methylated and unmethylated; in both cases, leukocyte DNA from healthy donors was used and, respectively, treated or untreated with CpG Methylase SssI (New England BioLabs) before the bisulfite modification. In addition, a non-template control (NTC), a reaction with water instead of a DNA template, was performed alongside each PCR run. Reaction products were analyzed electrophoretically in 3% agarose gel, stained with ethidium bromide, and visualized under UV illumination (GelDoc-It^®^310 Imaging system, Fisher Scientific, TFS) using visualization and analysis software VisionWorks^®^LS (UVP, Upland, CA, USA).

### 4.4. TP53 Mutation Analysis

*TP53* mutation status (exons 4–8) was evaluated by means of single-strand conformation analysis (SSCP; N = 84) and validated with Sanger sequencing (N = 29) and/or next-generation sequencing (NGS; N = 38). Mutation analysis was performed using IARC *TP53* (https://www.iarc.who.int/ (accessed on 20 July 2017)) [30] and The Catalogue of Somatic Mutations in Cancer (COSMIC; https://cancer.sanger.ac.uk/cosmic (accessed on 20 July 2017)) [31] databases.

Single-strand conformation polymorphism (SSCP) analysis was performed to analyze 5–9th *TP53* exons. DNA was amplified by PCR, using primers labeled with fluorescent dyes: a forward primer with 6-FAM (6-carboxyfluorescein) and reverse-with HEX (4,7,2’,4’,5’,7’-hexachloro-6-carboxyfluorescein). Final PCR mix volume was 20 µL and consisted of 200 ng DNA template, GeneAmp 10 × PCR Buffer, 25 nM MgCl_2_, 4 mM dNTP mix, 5U/µL AmpliTaq Gold^TM^ DNA polymerase (Applied Biosystems (ABI), TFS), 20 µM of each 6-FAM and HEX primers, DMSO, and deionized H_2_O. SSCP mix contained 0.5 µL PCR product, 0.5 µL GeneScan-500 LIZ size standard (ABI, TFS), and 15 µL HiDi formamide. T24 cell line DNA was used as a positive and healthy donor’s leukocyte DNA as a negative control. SSCP reactions were carried out on ABI PRISM 3130^®^ Genetic Analyzer and the results were analyzed by using GeneMapper^TM^ software (both from ABI, TFS), by which the displacements or alterations in the electropherogram peaks were recorded as mutations.

Sanger sequencing (SS) was used to confirm mutations detected by SSCP. Analyzed 5–9th *TP53* exons of SSCP-positive samples were first amplified by PCR, consisting of 200 ng DNA templates and the same reaction components as were used for SSCP analysis, which is described above. The sequencing reaction (20 µL), contained 5 µL PCR product, BigDye Terminator v3.1 Ready Reaction mix (ABI, TFS), 5× Sequencing Buffer, sense and antisense primers, and H_2_O. Sequencing reactions were carried out on ABI Prism 3130^®^ Genetic Analyzer and analyzed with SeqScape^TM^ software (ABI, TFS). Results were compared with reference *TP53* sequence from GenBank^®^ database. SSCP and SS methods were adapted from Holmila and Husgafvel-Pursiainen [32].

Next-generation sequencing (NGS) was performed using GS Junior 454 Sequencer (Roche Diagnostics by 454 Life science corp. Branford, CT, USA). A healthy female leukocyte DNA was used as a reference. All fragments were sequenced in both directions. DNA was amplified in 24 µL reaction mix, which contained 1x Phusion HF buffer, 0.2 mM of each dNTP, 0.3 µM of each primer, 0.5 U/µL HiFi Phusion polymerase, and 25 ng of DNA template (see details in Supplementary Table S5) Amplicons were purified with AMPure XP magnetic beads (TFS). Reaction products were fluorometrically analyzed using the Quant-It^TM^ PicoGreen dsDNA Assay kit (TFS) and the QuantiFluor system^®^ (Promega, Madison, WI, USA). Standard curve value was not less than R2 > 0.98. Emulsion PCR was performed using the emPCR Kit according to manufacturer’s instructions. Amplicons were mixed with capture beads using 10 uL of DNA library (at 1.33 molecules per bead concentration) for each forward and reverse strand amplification by emPCR and collected with the GS Junior Titanium emPCR Oil and Breaking Kit. For the sequencing procedure, The GS Titanium Sequencing Kit and GS Junior Titanium series protocol were followed (Roche). Sequencing data analysis was performed using GS Amplicon Variant Analyzer (AVA) (Roche). *TP53* sequence NC_000017.10 (NCBI37/hg19; Chr17:7571720…7590868) was used as the reference sequence (corresponding transcript and protein IDs are ENST00000269305.4 and P04637, respectively).

### 4.5. Statistical Analysis

The two-sided Fisher’s exact test was used for analysis of gene methylation status and other categorical clinical variables (for patients’ age, two groups of < 50 and > 50 yrs. were compared). Mann–Whitney testing was applied to continuous data. Cox proportional hazards regression (with backward variable selection) and Kaplan–Meier analysis (with multiple testing correction (Bonferroni) and additionally corrected p-values) were used to assess the associations between clinical parameters and survival. Calculations were performed by using GraphPad Prism 8.01 (GraphPad Software, Inc., San Diego, CA, USA) and MedCalc 12.7.0.0 (MedCalc Software Ltd., Ostend, Belgium). In all cases, *p* ≤ 0.05 was considered statistically significant.

## 5. Conclusions

DNA methylation of *RASSF1, PRKCB, APC*, and *RUNX3* significantly separates BC from noncancerous specimens and also is more frequently found in non-TN BC cases, while higher methylation frequency of *MT1E* and *FILIP1L* is associated with TN BC. The combination of two-to-three epigenetic biomarkers (*FILIP1L, RUNX3*, and *MT1E*) is an informative tool for BC-outcome predictions. Further investigations of these DNA methylation biomarkers are needed, especially for improved characterization of the TN BC subtype.

## Figures and Tables

**Table 1:** Clinical parameters of *TP53* mutation-positive (*TP53-mut*) and negative (*TP53-wt*) BC cases.

Variables	Characteristics	*TP53-mut*, N (%)	*TP53*-wt, N (%)	*p*-Value
BC patients, N		33	53	
Median age at BC diagnosis	yrs. (IQR)	55 (24)	61 (19)	0.377
Histological type	Ductal; N (%)	32 (38)	46 (55)	0.396
	Lobular; N (%)	1 (1)	5 (6)	
Pathological stage	T1; N (%)	15 (17)	24 (28)	1.0
	T2; N (%)	18 (21)	29 (34)	
Lymph node metastasis	No (N0); N (%)	21 (25)	25 (29)	0.186
	Yes (N1); N (%)	12 (14)	27 (32)	
Tumor differentiation grade	G1; N (%)	7 (8)	11 (13)	(G1 vs. G2) **0.032** (G1 vs. G3) 0.072. (G2 vs. G3) **< 0.0001**
	G2; N (%)	4 (5)	31 (36)	
	G3; N (%)	22 (26)	10 (12)	
Intrinsic BC subtype	LA; N (%)	5 (6)	31 (36)	(LA vs. LB) **0.010** (LA vs. HER2+) 0.073 (LA vs. TN) **< 0.0001** (LB vs. HER2+) 0.734 (LB vs. TN) **0.030** (HER2 + vs. TN) **0.010**
	LB; N (%)	9 (10)	10 (12)	
	Her2+; N (%)	6 (7)	10 (12)	
	TN; N (%)	13 (15)	2 (2)	
ER status	Negative; N (%)	15 (18)	4 (5)	**<0.0001**
	Positive; N (%)	18 (21)	48 (56)	
PR status	Negative; N (%)	16 (19)	12 (14)	**0.018**
	Positive; N (%)	17 (20)	41 (48)	
HER2 status	Negative; N (%)	28 (33)	43 (50)	0.775
	Positive; N (%)	5 (6)	10 (12)	
Ki-67	Negative; N (%)	8 (9)	39 (46)	**<0.0001**
	Positive; N (%)	24 (28)	14 (16)	
Survival	Remission; N (%)	18 (31)	24 (41)	0.773
	Death; N (%)	6 (10)	10 (17)	

Abbreviations: ER—estrogen receptor; G1—good, G2—moderate, G3—poor differentiation grade; HER2—human epidermal growth factor receptor 2; HER2+—HER2 positive and Luminal B HER2 positive; IQR—interquartile range; LA—Luminal A; LB—Luminal B; N—lymph node affection; PR—progesterone receptor; T—tumor stage; *TP53-mut*—*TP53* mutated and *TP53-wt—wild type TP53* gene status; TN—triple negative BC. The bolded *p* < 0.05.

**Table 2:** Univariate and multivariate Cox proportional hazards regression analysis for overall survival.

Covariate		Univariate Analysis		Multivariate Analysis	
		HR (95% CI)	*p*-Value	HR (95% CI)	*p*-Value
Methylated: yes/no	*FILIP1L*	3.33 (0.92−11.97)	0.067		
	*P16*	3.14 (0.72−13.67)	0.128		
	*RUNX3*	2.07 (0.7−5.50)	0.147	4.64 (1.15−18.75)	**0.032**
	*P14*	2.14 (0.29−16.03)	0.462		
	*MT1F*	2.02 (0.27−15.15)	0.497		
	*ADAMTS12*	1.42 (0.51−3.98)	0.502		
	*DAPK1*	1.34 (0.39−4.62)	0.643		
	*MT1E*	1.20 (0.47−3.11)	0.704		
	*NAALAD2*	1.15 (0.43−3.11)	0.779		
Clinical–pathological charact.	Age (cont.)	1.04 (1.00−1.09)	**0.049**	1.07 (1.02−1.12)	**0.010**
	T (1 vs. 2)	1.06 (0.42−2.69)	0.897		
	N (yes/no)	2.15 (0.84−5.53)	0.113		
	G (≤2 vs. 3)	1.55 (0.62−3.88)	0.357		
	TN subtype (yes/no)	2.91 (1.04−8.18)	**0.044**	13.92 (2.97−65.20)	**0.010**
	Molecular biomarkers (high/low):				
	ER	0.61 (0.22−1.71)	0.349		
	PR	0.68 (0.26−1.74)	0.422		
	HER2	0.25 (0.03−1.86)	0.177		
	Ki-67	1.02 (0.40−2.58)	0.963		
	*TP53-mut* (yes/no)	0.95 (0.34−2.65)	0.919		

Abbreviations: CI: confidence interval; ER—estrogen receptor; G1—good, G2—moderate, G3—poor differentiation grade; HER2—human epidermal growth factor receptor 2; HR: hazard ratio; N—lymph node affection; PR—progesterone receptor; T—tumor stage; *TP53-mut*—*TP53* mutated; TN—triple-negative BC. Underlined are variables included in multivariate analysis. The bolded *p* < 0.05.

**Table 3:** Demographic and clinical–pathological characteristics of breast cancer (BC) patients distributed by breast cancer (BC) subtypes.

BC Features		BC Subtypes				
		LA	LB	HER2+ *	TN	Total BC Cases
		N = 55 (%)	N = 24 (%)	N = 21 (%)	N = 16 (%)	N = 116 (%)
Median age at BC diagnosis	yrs. (IQR)	61 (19)	58 (22)	55 (23)	52 (26)	
Histology	Ductal; N (%)	45 (82)	22 (92)	18 (86)	16 (100)	101 (87)
	Lobular; N (%)	8 (15)	2 (8)	3 (14)	0 (0)	13 (11)
	Apocrine; N (%)	2 (3)	0 (0)	0 (0)	0 (0)	2 (2)
Tumor stage	T1; N (%)	33 (60)	13 (54)	11 (52)	6 (38)	63 (54)
	T2; N (%)	22 (40)	11 (46)	10 (48)	10 (63)	53 (46)
Spread to lymph nodes	N0; N (%)	33 (54)	13 (54)	11 (52)	10 (63)	67 (58)
	N1; N (%)	21 (61)	11 (46)	10 (48)	6 (38)	48 (42)
Grade	G1; N (%)	16 (29)	3 (13)	2 (10)	2 (13)	23 (20)
	G2; N (%)	35 (64)	7 (29)	9 (43)	1 (6)	52 (45)
	G3; N (%)	4 (7)	14 (58)	10 (48)	13 (81)	41 (35)
Ki-67 expression	Ki-67 neg; N (%)	53 (96)	0 (0)	15 (71)	0 (0)	68 (59)
	Ki-67 pos; N (%)	2 (4)	24 (100)	6 (29)	16 (100)	48 (41)
Survival (N = 78)	Remission; N (%)	25 (71)	13 (68)	12 (92)	6 (55)	56 (72)
	Death; N (%)	10 (29)	5 (26)	1 (8)	5 (45)	21 (27)
	Relapse; N (%)	0 (0)	1 (5)	0 (0)	0 (0)	1 (1)
	5-year survival (%)	25 (71)	16 (94)	11 (92)	6 (55)	58 (74)

* LBHER2 and HER2; the count is 16 and 5, respectively. Abbreviations: G1—good, G2—moderate, G3—poor differentiation; HER2+—HER2 positive and Luminal B HER2 positive; IQR—interquartile range; LA—Luminal A; LB—Luminal B; N—lymph node affection; T—tumor stage; TN—triple-negative BC.

## Data Availability

The datasets analyzed during the current study are available from the corresponding author upon reasonable request.

## Supplementary Materials

The supporting information can be downloaded at: https://www.mdpi.com/article/10.3390/ijms232315429/s1.
