# Supplementary material for: Analysis of Intrinsic Breast Cancer Subtypes: The Clinical Utility of Epigenetic Biomarkers and TP53 Mutation Status in Triple-Negative Cases
Source: Int J Mol Sci. 2022 Dec 6;23(23):15429. doi: 10.3390/ijms232315429 (PMC9741387; doi:10.3390/ijms232315429)
Supplement: Supplementary file 1 [file ijms-23-15429-s001.zip › Supplementary Table S1.pdf]

Supplementary Table S1. Methylation frequencies of analysed genes in breast cancer (BC) tumors, control samples and BC subtypes.

Gene	Methylated BC (n/N)	Sensitivity, %	Methylated controls (n/N)	Specificity, %	P-value	LA, % (n/N)	LB, % (n/N)	HER2+, % (n/N)	TN, % (n/N)
PRKCB	86/111	77	0/29	100	**< 0.0001**	85 (46/54)	83 (19/23)	83 (15/18)	38 (6/16)
RASSF1	79/115	69	4/29	86	**< 0.0001**	78 (43/55)	65 (15/23)	67 (14/21)	44 (7/16)
ADAMTS12	67/107	63	2/29	93	**< 0.0001**	65 (34/52)	68 (15/22)	71 (12/17)	38 (6/16)
APC	61/115	53	0/29	100	**< 0.0001**	56 (30/54)	50 (12/24)	71 (15/21)	25 (4/16)
RUNX3	46/114	40	0/29	100	**< 0.0001**	41 (22/54)	48 (11/23)	57 (12/21)	6 (1/16)
ESR1-1	51/116	44	2/29	93	**0.0002**	47 (26/55)	50 (12/24)	38 (8/21)	36 (5/14)
MGMT	28/116	24	0/28	100	**0.002**	22 (12/55)	25 (6/24)	29 (6/21)	25 (4/16)
GSTP1	24/116	21	0/29	100	**0.004**	20 (11/55)	21 (5/24)	33 (7/21)	6 (1/16)
MT1E	40/113	35	4/29	86	**0.026**	31 (17/55)	39 (9/23)	21 (4/19)	63 (10/16)
RARB	22/116	19	1/29	97	**0.046**	18 (10/55)	25 (6/24)	14 (3/21)	19 (3/16)
ESR1-4	63/111	57	11/29	62	0.095	57 (30/53)	63 (15/24)	65 (13/20)	36 (5/14)
MT1G	39/112	35	6/29	79	0.184	37 (20/54)	45 (10/22)	35 (7/20)	13 (2/16)
DAPK1	17/116	15	7/29	76	0.262	16 (9/55)	13 (3/24)	19 (4/21)	6 (1/16)
FILIP1L	53/102	52	18/28	36	0.288	42 (21/50)	57 (12/21)	38 (6/16)	93 (14/15)
NAALAD2	61/108	56	13/28	54	0.401	56 (29/52)	64 (14/22)	50 (9/18)	56 (9/16)
P16	4/116	3	0/29	100	0.584	2 (1/55)	8 (2/24)	5 (1/21)	0 (0/16)
MT1F	2/110	2	0/29	100	1.0	4 (2/54)	0 (0/22)	0 (0/18)	0 (0/16)
P14	3/115	3	0/29	100	1.0	4 (2/54)	4 (1/24)	0 (0/18)	0 (0/16)

Abbreviations: LA – Luminal A, LB – Luminal B, HER2+ – HER2 positive and Luminal B HER2 positive, TN BC – triple negative BC.
