# Supplementary material for: Analysis of Intrinsic Breast Cancer Subtypes: The Clinical Utility of Epigenetic Biomarkers and TP53 Mutation Status in Triple-Negative Cases
Source: Int J Mol Sci. 2022 Dec 6;23(23):15429. doi: 10.3390/ijms232315429 (PMC9741387; doi:10.3390/ijms232315429)
Supplement: Supplementary file 1 [file ijms-23-15429-s001.zip › Supplementary Table S2.pdf]

Supplementary Table S2. Pathogenic *TP53* pathogenic gene variants identified in breast cancer.

Nr.	Mutation site, bp	Exon / Intron	Codon no	Sequence alteration	Mutation type	WT aminoacid	Mutant aminoacid	Mutant protein codon	Mutation effect	Affected gene region	Average mutation frequency, %*	Mutation found in samples
1	11608:T>C	4-intron	0	c.375+2T>C	A:T>G:C	-	-	-	splice site alteration	intron	45.24	1
2	11608:T>G	4-intron	0	c.375+2T>G	A:T>C:G	-	-	-	splice site alteration	intron	58.8	1
3	11621-11643:DEL(23)	4-intron	0	c.376-14_384del23	del	-	-	-	splice site alteration	intron	58.8	1
4	12278:T>C	4-intron	0	c.376-86T>C	A:T>G:C	-	-	-	intronic	intron	10.19	1
5	12383:A>G	5-exon	132	c.395A>G	A:T>G:C	Lys	Arg	p.K132R	missense	DNA binding	49.34	1
6	12457:G/T	5-exon	157	c.469G>T	G:C>T:A	Val	Phe	p.V157F	missense	DNA binding	44.43	1
7	12515:G/A	5-exon	176	c.527G>A	G:C>A:T	Cys	Tyr	p.C176Y	missense	DNA binding	33.75	1
8	12517-12534:DEL(18)	5-exon	177	c.529_546del18	del	Pro	-	p.P177_C182delPHHERC	frameshift	DNA binding	45.05	1
9	12521:A/C	5-exon	178	c.533A>C	A:T>C:G	His	Pro	p.H178P	missense	DNA binding	0.53	2
10	12524:A>G	5-exon	179	c.536A>G	A:T>G:C	His	Arg	p.H179R	missense	DNA binding	53.99	1
11	12643:C>T	6-exon	192	c.574C>T	G:C>A:T	Gln	STOP	p.Q192*	nonsense	DNA binding	75.63	1
12	12650:T>A	6-exon	194	c.581T>A	A:T>T:A	Leu	His	p.L194H	missense	DNA binding	20.98	1
13	12653:T>C	6-exon	195	c.584T>C	A:T>G:C	Ile	Thr	p.I195T	missense	DNA binding	44.97	1
14	12655:C>T	6-exon	196	c.586C>T	G:C>A:T at CpG	Arg	STOP	p.R196*	nonsense	DNA binding	56.21	1
15	12694-12695:AG>--	6-exon	209	c.625_626del2	del	Arg	-	-	frameshift	DNA binding	18.41	1
16	12710:A>G	6-exon	214	c.641A>G	A:T>G:C	His	Arg	p.H214R	missense	DNA binding	76.55	1
17	13380:G>A	7-exon	248	c.743G>A	G:C>A:T at CpG	Arg	Gln	p.R248Q	missense	DNA binding	46.2	1
18	13392:T/C	7-exon	252	c.755T>C	A:T>G:C	Leu	Pro	p.L252P	missense	DNA binding	53.86	1
19	13420:G>A	7-intron	0	c.782+1G>A	G:C>A:T	-	-	-	splice site alteration	intron	71.41	1
20	13781:G/-	8-exon	267	c.801del1	del	Arg	-	-	frameshift	DNA binding	34.5	1
21	13797:C/T	8-exon	273	c.817C>T	G:C>A:T at CpG	Arg	Cys	p.R273C	missense	DNA binding	43.04	1
22	13798:G>A	8-exon	273	c.818G>A	G:C>A:T at CpG	Arg	His	p.R273H	missense	DNA binding	90.78	2
23	13813:C>G	8-exon	278	c.833C>G	G:C>C:G	Pro	Arg	p.P278R	missense	DNA binding	30.53	1
24	13833:G>A	8-exon	285	c.853G>A	G:C>A:T	Glu	Lys	p.E285K	missense	DNA binding	36.89	1
25	13833:G>T	8-exon	285	c.853G>T	G:C>T:A	Glu	STOP	p.E285*	nonsense	DNA binding	16.26	1
26	13896:C/T	8-exon	306	c.916C>T	G:C>A:T at CpG	Arg	STOP	p.R306*	nonsense	DNA binding	29.64	1
27	13900:G/T	8-intron	0	c.919+1G>T	G:C>T:A	-	-	-	splice site alteration	intron	32.28	1

*- Average mutation frequency calculated as forward and reverse DNA chain analysis by Roche Junior Sequencer Software.
