# Supplementary material for: Analysis of Intrinsic Breast Cancer Subtypes: The Clinical Utility of Epigenetic Biomarkers and TP53 Mutation Status in Triple-Negative Cases
Source: Int J Mol Sci. 2022 Dec 6;23(23):15429. doi: 10.3390/ijms232315429 (PMC9741387; doi:10.3390/ijms232315429)
Supplement: Supplementary file 1 [file ijms-23-15429-s001.zip › Supplementary Table S3.pdf]

Supplementary Table S3. Primers used for methylation-specific PCR.

Gene	Primer pair ID	Forward 5'-3'	Reverse 5'-3'	Amplicon length, bp	PCR cycles	Primer annealing T, (°C)	Ref.
PRKCB	M	TAAGCGTAGTTGGACGAGC	AAAACGACGACCGCTACTAC	124	36	56	[27]
	U	TGTTAAGTGTAGTTGGATGAGT	AAAACAACAACCACTACTACACC	127			
ADAMTS12	M	GAGTTCGGGAGGAAGATGTATC	ACAACGACTACAAAACTACCCG	241	35	62	
	U	GAGTTTGGGAGGAAGATGTATT	AAACAACAACTACAAAACTACCA	243			
NAALAD2	M	TATTTATTATGTTCGGGTTATTGC	CCTACTACATTCGCGAACTTC	244	35	58	
	U	GTTATTTATTATGTTTGGGTTATTGT	CCTACTACATTCACAAACTTCAA	246			
FILIP1L	M	TACGGTTCGTTTATACGGTC	CGACCTATAAACGTTACGTCA	160	37	57	
	U	GGAATTATGGTTTGTTTATATGGTT	CCCAACCTATAAACATTACATCAC	167			
DAPK1	M	GGATAGTCGGATCGAGTTAACGTC	CCCTCCCAAACGCCGA	98	37	66	[28]
	U	GGAGGATAGTTGGATTGAGTTAATGTT	CAAATCCCTCCCAAACACCAA	106			
GSTP1	M	TTCGGGGTGTAGCGGTCGTC	GCCCCAATACTAAATCACGACG	91	37	60	
	U	GATGTTTGGGGTGTAGTGGTTGTT	CCACCCCAATACTAAATCACAACA	97			
MGMT	M	ATTTGGTGAGTGTTTGGGTCGTTTC	AAAACGCACCTAAAACTCGCCC	159	37	67	
	U	ATTTGGTGAGTGTTTGGGTTGTTTT	AAAACACACCTAAAACTCACCC	159		64	
P14	M	GTGTTAAAGGGCGGCGTAGC	AAAACCCTCACTCGCGACGA	122	37	66	
	U	TTTTTGGTGTTAAAGGGTGGTGTAGT	CACAAAAACCCTCACTCACAACAA	132			
P16	M	TTATTAGAGGGTGGGGCGGATCGC	GACCCCGAACCGCGACCGTAA	150	36	64-30s	
	U	TTATTAGAGGGTGGGGTGGATTGT	CAACCCCAAACCACAACCATAA	151			
RARB	M	TCGAGAACGCGAGCGATTCG	GACCAATCCAACCGAAACGA	145	39	60	
	U	TTGAGAATGTGAGTGATTTGA	AACCAATCCAACCAAAACAA	145			
RASSF1	M	GGGTTTTGCGAGAGCGCG	GCTAACAAACGCGAACCG	169	39	61	
	U	GGTTTTGTGAGAGTGTGTTTAG	CACTAACAAACACAAACCAAAC	169		63	
ESR1-1	M	TTTTGGGATTGTATTTGTTTTCGTC	AACAAAATACAAACCGTATCCCCG	197	37	60	
	U	TTTTGGGATTGTATTTGTTTTTGTTG	AAACAAAATACAAACCATATCCCCA	198			
ESR1-4	M	CGAGTTGGAGTTTTTGAATCGTTC	CTACGCGTTAACGACGACCG	151	37	58	
	U	ATGAGTTGGAGTTTTTGAATTGTTT	ATAAACCTACACATTAACAACAACCA	158			
MT1E	M	GGATTTCGGGAATATCGC	ACGAAAATCGAACCGAAC	217	38	56	[29]
	U	TTTGGATTTTGGGAATATTGT	ACAAAAATCAAACCAAACACA	220			
MT1F	M	GTATTCGGAATTTTAAGGGGC	CGAACCGTCCCTTTAAAATC	134	36	56	
	U	TAGGTATTTGGAATTTTAAGGGGT	CACAAACCATCCCTTTAAAATC	139			
MT1G	M	TCGTATACGGGGGGTATAGC	GCGATCCCGACCTAAACT	131	37	58	
	U	AAGTTGTATATGGGGGGTATAGT	CCCACAATCCCAACCTAAACT	137			
APC	M	TATTGCGGAGTGCGGGTC	TCGACGAACTCCCGACGA	98	38	59	Methyl Primer Express v1.0 software
	U	GTGTTTTATTGTGGAGTGTGGGTT	CCAATCAACAAACTCCCAACAA	108			Methyl Primer Express v1.0 software
RUNX3	M	ATAATAGCGGTCGTTAGGGCGTCG	GCTTCTACTTTCCCGCTTCTCGCG	115	38	59	Methyl Primer Express v1.0 software
	U	ATAATAGTGGTTGTTAGGGTGTTG	ACTTCTACTTTCCCACTTCTCACA	115			Methyl Primer Express v1.0 software

Abbreviations: M - methylated sequence, U - unmethylated sequence
