# Supplementary material for: Analysis of Intrinsic Breast Cancer Subtypes: The Clinical Utility of Epigenetic Biomarkers and TP53 Mutation Status in Triple-Negative Cases
Source: Int J Mol Sci. 2022 Dec 6;23(23):15429. doi: 10.3390/ijms232315429 (PMC9741387; doi:10.3390/ijms232315429)
Supplement: Supplementary file 1 [file ijms-23-15429-s001.zip › Supplementary Table S4.pdf]

Supplementary Table S4. Methylation specific PCR cycling conditions

**Temperature, ºC**	**Duration**	**Cycles**
95 ºC	7 min	1
95 ºC	45 s	35–37
According to gene*	45 s	
72 ºC	45 s	
72 ºC	10 min	1
4 ºC	∞	1

* *P14* – 66 ºC; *P16* – 64 ºC–30s; *MGMT* – 67 ºC (M) and 64 ºC (U); *RARB* – 60 ºC; *RASSF1* – 61 ºC (M) and 63 ºC (U); *DAPK1* – 66 ºC; *GSTP1* – 60 ºC; *ESR1-1* – 60 ºC; *ESR1-4* – 58 ºC; *PRKCB* – 56 ºC; *MT1E* – 56 ºC; *MT1F* – 55 ºC; *MT1G* – 58 ºC; *APC* – 59 ºC; *ADAMTS12* – 62 ºC; *RUNX3* – 59 ºC; *NAALAD2* – 58 ºC.
