# Supplementary material for: Analysis of Intrinsic Breast Cancer Subtypes: The Clinical Utility of Epigenetic Biomarkers and TP53 Mutation Status in Triple-Negative Cases
Source: Int J Mol Sci. 2022 Dec 6;23(23):15429. doi: 10.3390/ijms232315429 (PMC9741387; doi:10.3390/ijms232315429)
Supplement: Supplementary file 1 [file ijms-23-15429-s001.zip › Supplementary Table S5.pdf]

Supplementary Table S5

PCR cycling conditions for *TP53* amplicon library creation for NGS

Temperature, ºC	Duration	Cycles
98 ºC	30 s	1
98 ºC	10 s	34
65 ºC	30 s	
72 ºC	30 s	
72 ºC	10 min	1
4 ºC	∞	1

NGS Primers used for *TP53* amplicon library PCR

Primer ID	Primer	Primer sequence (5‘-3‘)
Exon - 4	Forward	uni-MID-GACTGCTCTTTTCACCCATCTA
	Reverse	uni-MID-AGGAATCCCAAAGTTCCAAACAA
Exon - 5	Forward	uni-MID-TTGCAGGAGGTGCTTACG
	Reverse	uni-MID-GAGCAATCAGTGAGGAATCAGA
Exon - 6	Forward	uni-MID-GTCACAGCACATGACGGAG
	Reverse	uni-MID-GGTTATAGGGAGGTCAAATAAGCAG
Exon - 7	Forward	uni-MID-CTTGCCACAGGTCTCCC
	Reverse	uni-MID-AGAAGCCACAGGTTAAGAGGT
Exon - 8	Forward	uni-MID-CTTTGGGACCTCTTAACCTGT
	Reverse	uni-MID-TGATAAAAGTGAATCTGAGGCATAACT

Abbrevations: uni - universal segment; MID - multiplex identifier.
